# Targeting Methyltransferases in Human Pathogenic Bacteria: Insights into Thymidylate Synthase (TS) and Flavin-Dependent TS (FDTS)

**DOI:** 10.3390/molecules24081638

**Published:** 2019-04-25

**Authors:** Cecilia Pozzi, Ludovica Lopresti, Giusy Tassone, Stefano Mangani

**Affiliations:** Department of Biotechnology, Chemistry and Pharmacy–Department of Excellence 2018-2020, University of Siena, via Aldo Moro 2, 53100 Siena, Italy; lopresti4@student.unisi.it (L.L.); giusy.tassone@unisi.it (G.T.)

**Keywords:** thymidylate synthase, flavin-dependent thymidylate synthase, mechanism of action, half-site reactivity, inhibitors, selectivity

## Abstract

In cells, thymidylate synthases provide the only *de novo* source of 2′-deoxythymidine-5′-monophosphate (dTMP), required for DNA synthesis. The activity of these enzymes is pivotal for cell survival and proliferation. Two main families of thymidylate synthases have been identified in bacteria, folate-dependent thymidylate synthase (TS) and flavin-dependent TS (FDTS). TS and FDTS are highly divergent enzymes, characterized by exclusive catalytic mechanisms, involving different sets of cofactors. TS and FDTS mechanisms of action have been recently revised, providing new perspectives for the development of antibacterial drugs targeting these enzymes. Nonetheless, some catalytic details still remain elusive. For bacterial TSs, half-site reactivity is still an open debate and the recent evidences are somehow controversial. Furthermore, different behaviors have been identified among bacterial TSs, compromising the definition of common mechanisms. Moreover, the redox reaction responsible for the regeneration of reduced flavin in FDTSs is not completely clarified. This review describes the recent advances in the structural and functional characterization of bacterial TSs and FDTSs and the current understanding of their mechanisms of action. Furthermore, the recent progresses in the development of inhibitors targeting TS and FDTS in human pathogenic bacteria are summarized.

## 1. Introduction

Thymidylate synthase is a class of methyltransferase enzymes required for *de novo* 2′-deoxythymidine-5′-monophosphate (dTMP) synthesis. These enzymes catalyze the methylation of 2′-deoxyuridine-5′-monophosphate (dUMP) using *N*^5^,*N*^10^-methylentetrahydrofolate (CH_2_H_4_folate) as co-substrate. Thymidylate synthases are pivotal for cell survival and replication since they provide the unique biosynthetic source of dTMP, essential for DNA synthesis.

**Figure 1:**
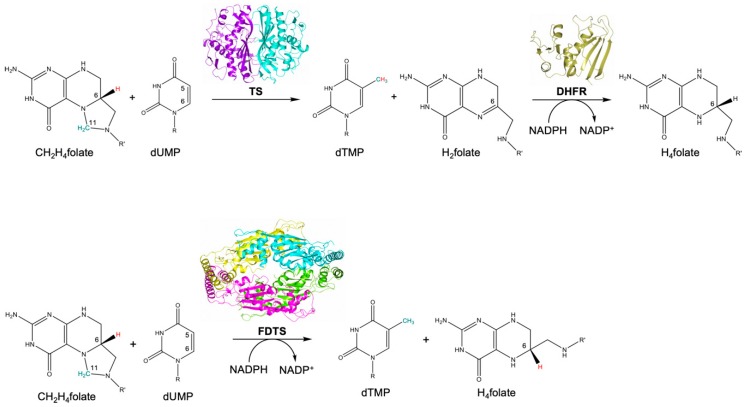
Reactions catalyzed by TS and DHFR (upper panel) and FDTS (lower panel) (TS, PDB id 3QJ7; DHFR, PDB id 5UIH; FDTS, PDB id 3GCW). In the FDTS catalyzed reaction, the cofactor FAD is not displayed because it is oxidized and subsequently reduced in each catalytic cycle. R = 2′-deoxyribose-5′-monophosphate; R’ = *p*-benzoyl-amino-l-glutamic acid.

In bacteria, two main families of thymidylate synthases are known, folate-dependent thymidylate synthase (TS, EC 2.1.1.45) and flavin-dependent thymidylate synthase (FDTS, EC 2.1.1.148), encoded by *thyA* and *thyX* genes, respectively [1,2]. TS and FDTS are highly divergent at all structural levels [1,2]. These enzymes are also characterized by exclusive catalytic mechanisms that involve different sets of cofactors [1,2,3,4]. At variance with TS that relies only on CH_2_H_4_folate, FDTS requires CH_2_H_4_folate, flavin adenine dinucleotide (FAD) and nicotinamide adenine dinucleotide phosphate (NADPH) to perform its action [1,2,3,4]. In the TS-catalyzed reaction, CH_2_H_4_folate provides both the methylene group and the hydride required to convert dUMP in dTMP (Figure 1) [1,5]. Dihydrofolate (H_2_folate), generated as byproduct of the TS reaction, is then converted to tetrahydrofolate (H_4_folate) through a second enzyme, dihydrofolate reductase (DHFR, encoded by *folA* gene) (Figure 1) [5]. On the other hand, FDTSs are able to combine the TS and DHFR functions, relying on the two additional cofactors, NADPH and FAD (Figure 1) [2]. FDTSs use CH_2_H_4_folate solely as the methyl donor, yielding H_4_folate (Figure 1) [2,4]. At a later stage, the pathways of TS and FDTS converge in the recycling of the cofactor CH_2_H_4_folate from H_4_folate, ensured by the enzyme serine hydroxymethyltransferase [5].

At variance with TS that is present in some viruses and various organisms including humans, FDTS is unique to bacteria [1,2,3,4]. Important human pathogens, including *Helicobacter pylori*, *Borrelia Burgdorferi*, *Treponema pallidum*, *Chlamydia* species and *Rickettsia* species, rely only on FDTS for dTMP biosynthesis [2,6,7]. On the other hand, human pathogenic bacteria such as *Staphylococcus aureus*, *Enterococcus faecalis* and *Pseudomonas aeruginosa*, have only the *thyA* gene, expressing solely the TS enzyme [2,6,7]. A third group of bacteria, possessing both *thyA* and *thyX* genes, has been identified [2,6,7]. *Bacillus anthracis*, *Clostridium botulinum*, and *Mycobacterium* species are examples of important human pathogens belonging to this group [2,6,7]. In view of their common biological function, the reason concomitant expression of TS and FDTS occurs in these bacteria is not yet fully understood. Studies on *Mycobacterium tuberculosis* have evidenced that the *thyX* gene is essential, while the *thyA* deletion confers *p*-aminosalicylic acid resistance [6]. Furthermore, investigations on multi-drug resistant strains of *Mycobacterium tuberculosis*, have shown up-regulation of the *thyX* gene, responsible for FDTS overexpression [8].

Nowadays, the widespread diffusion of antibiotic resistance is an important health issue [9,10,11,12]. The major challenges are the identification of new microbial targets and the development of effective antibiotic therapies able to treat resistant infections. For this purpose, FDTS represents a promising target for the development of new antibiotics, since it has no counterpart enzyme in the human host [13,14]. On the other hand, TS is highly conserved in human and bacteria creating limitations for the development of inhibitors selectively targeting the bacterial enzyme [15]. Recent studies have provided important new insights into the catalytic process of both methyltransferase enzymes [3,4]. Indeed, new mechanisms of action for TS and FDTS have been recently proposed [3,4], opening new perspectives for the development of antibacterial drugs targeting these enzymes. This review is aimed to summarize the current understanding of structure and function of bTSs and FDTSs and the recent progresses in the development of inhibitors targeting these enzymes in human pathogenic bacteria.

## 2. Bacterial Thymidylate Synthases (bTSs)

### 2.1. Structural Insights into bTSs from Human Pathogens

Few crystallographic structures of TSs from human pathogenic bacteria have been reported to date. The structures of TSs from *Mycobacterium tuberculosis* (*Mtb*TS; PDB id 3QJ7, unpublished research), *Staphylococcus aureus* (*Sa*TS; PDB id 4DQ1, unpublished research), *Enterococcus faecalis* (*Ef*TS; PDB id 3UWL [16]), *Escherichia coli* (*Ec*TS; PDB id 1F4B [17]), *Brucella melitensis* (*Bm*TS; PDB id 3IX6, unpublished research), and *Elizabethkingia anophelis* (*Ea*TS; PDB id 6AUJ, unpublished research) are currently available in the Protein Data Bank (PDB), but limited information is reported in the literature.

**Figure 2:**
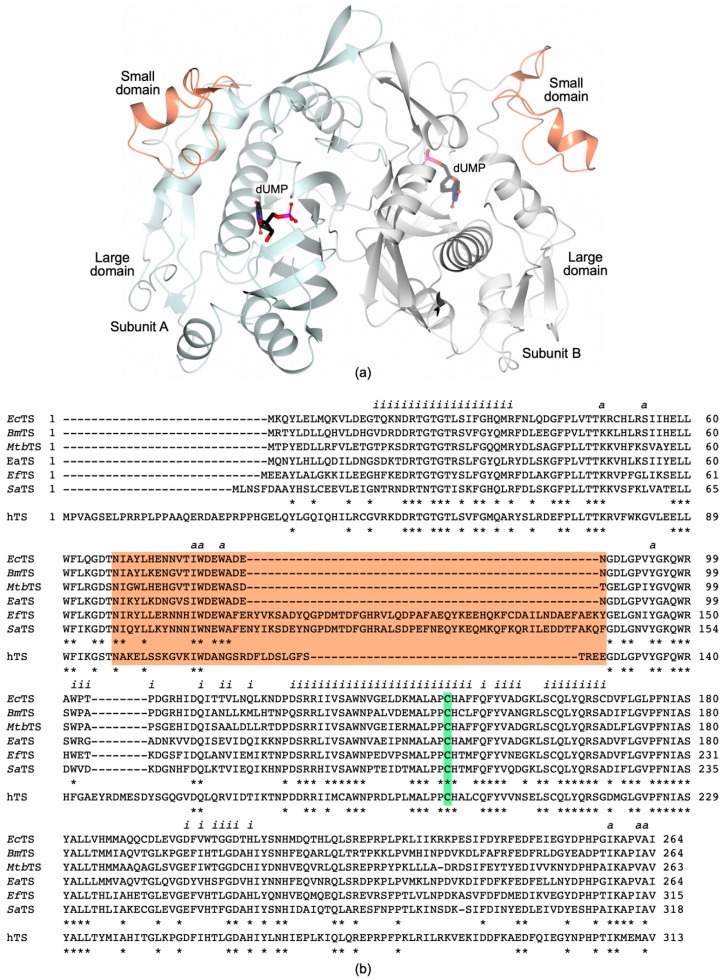
(**a**) Cartoon representation of the TS homodimer (*Mtb*TS, PDB id 3QJ7, unpublished research). The LD of Subunits A and B are colored light cyan and white, respectively. The SD (residues 67–87, in *Mtb*TS) is colored coral in both subunits. The position of the active site is indicated by the presence of the substrate dUMP (in sticks, black carbons). (**b**) Sequence alignment of TSs from pathogenic bacteria and the human enzyme (hTS). *Enterococcus faecalis* TS (*Ef*TS, UniProtKB: Q834R3), *Staphylococcus aureus* TS (*Sa*TS, UniProtKB: P67046), *Brucella melitensis* TS (*Bm*TS, UniProtKB: P67042), *Escherichia coli* TS (*Ec*TS, UniProtKB: P0A884), *Mycobacterium tuberculosis* TS (*Mtb*TS, UniProtKB: P9WFR9), and *Elizabethkingia anophelis* TS (*Ea*TS, UniProtKB: A0A077EAN3); human TS (hTS, UniProtKB: P04818). The present alignment includes only the TS sequences from human pathogenic bacteria for which the structure has been characterized. The SD is highlighted in coral and the catalytic cysteine in green. Conserved residues are indicated by asterisks. Residues forming the dimer interface and the active site are indicated by “*i*” and “*a*”, respectively.

**Figure 3:**
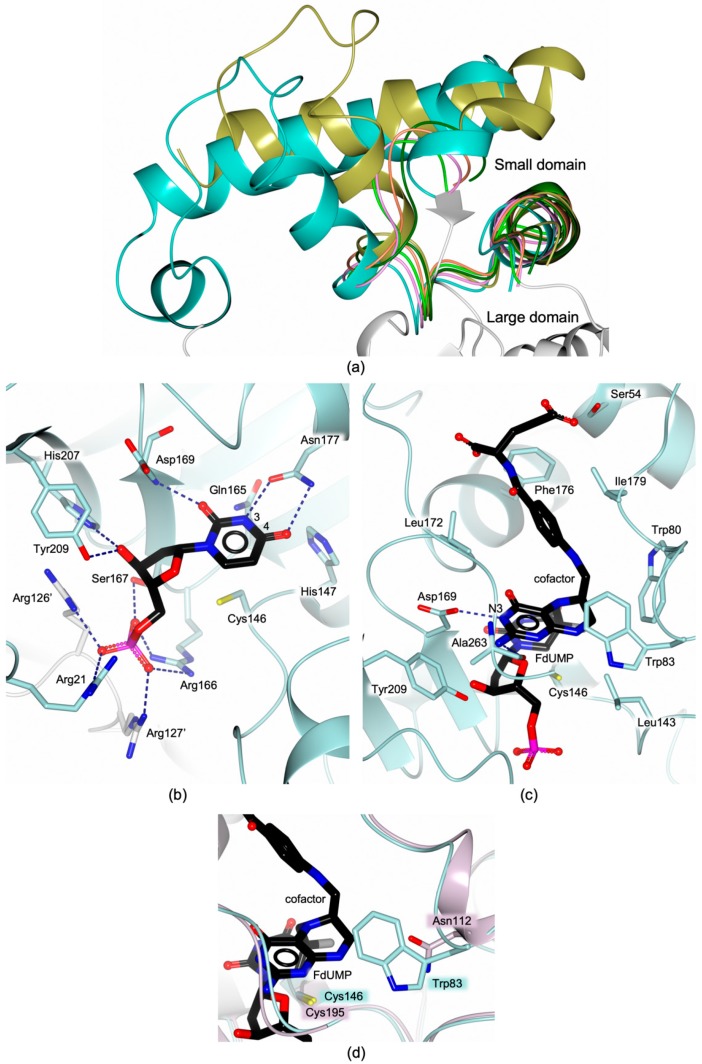
(**a**) Superimposition among the SD of *Mtb*TS (PDB id 3QJ7, unpublished research, coral trace), *Sa*TS (PDB id 4DQ1, unpublished research, gold trace), *Ef*TS (PDB id 3UWL [16], cyan trace), *Ec*TS (PDB id 1F4B [17], green trace), *Bm*TS (PDB id 3IX6, unpublished research, dark green trace), and *Ea*TS (PDB id 6AUJ, unpublished research, pink trace). (**b**) Conserved dUMP interactions within the substrate binding pocket of the TS active site (*Ec*TS sequence numbering). (**c**) Cofactor interactions within the active site (*Ec*TS sequence numbering). Only direct interactions are shown in Panels b and c for clarity. (**d**) Active site view of the superimposition of bTS and hTS, evidencing the presence of an unshared residue in the cofactor site, namely Asn112 in hTS that replaces the conserved tryptophan residue present in bTSs (Trp83 in *Ec*TS). In all figures, oxygen atoms are colored red, nitrogen blue, sulfur yellow, phosphorous magenta and halogen grey.

TS works as an obligate homodimer since residues from both subunits contribute to create the enzyme active site. Each subunit is composed of two domains, named large and small domains (LD and SD, respectively, Figure 2a). The LD, representing the conserved core of the enzyme, has a mixed α/β structure characterized by seven α-helices and eight β-strands (Figure 2a). A five-stranded β-sheet in the LD of each subunit generates the dimer interface, a crucial area for the enzyme function and for inter-subunit communication [18,19,20]. On the other hand, the SD is highly variable among bTSs, in terms of amino acid composition and length (Figure 2b and Figure 3a). The active site is a shallow cavity embedded between the two domains, where the substrate and the cofactor bind in a sequential order, relying on dUMP binding followed by CH_2_H_4_folate. A recent study has evidenced that the binding order of substrate and cofactor is tightly controlled in *Ec*TS, whereas it is less stringent for the human enzyme (hTS) [21].

The substrate binding pocket is highly conserved among bTSs (Figure 2b and Figure 3b). The dUMP phosphate moiety is bound to four conserved arginine residues (Arg21, Arg166, Arg126′ and Arg127′ in *Ec*TS), two of them protruding in the active site from the cognate subunit (Arg126′ and Arg127′ in *Ec*TS, Figure 3b). The four-arginine cluster is pivotal to anchor the substrate in its pocket, indeed substitution of these arginine residues impairs the enzyme catalytic activity [22,23,24,25]. This is also confirmed by the recent evidence of poor catalytic efficiency displayed by the TS from the non-pathogenic bacterium *Vibrio parahaemolyticus*, where Arg127′ is constitutively missing [26]. The correct orientation of the dUMP uracil ring is ensured by a conserved asparagine (Asn177 in *Ec*TS) [27]. The asparagine amide moiety forms two H-bonds with the nitrogen N3 (position 3) and the ketone oxygen in position 4, on the dUMP uracil (Figure 3b). Studies performed on *Ec*TS have revealed that the removal of the asparagine side chain disorganizes the substrate placement, drastically reducing the enzyme catalytic activity [27]. The correct orientation of the substrate is further ensured by the H-bonds between the dUMP ribose hydroxyl and conserved tyrosine and histidine residues (Tyr209 and His207 in *Ec*TS; Figure 3b). Recent evidence on *Ec*TS has shown that this conserved tyrosine is pivotal to pre-organize the active site for the hydride transfer in the last stages of the TS catalyzed reaction (the reaction mechanism is detailed in Section 2.2) [28].

The cofactor binding site is also shared among bacterial TSs (Figure 2b and Figure 3c). The pteridine nitrogen N3 is H-bonded to the carboxylate group of a conserved aspartic residue (Asp169 in *Ec*TS; Figure 3c). Within the bTS active site, the cofactor is mainly stabilized by hydrophobic interactions, indeed it entails a network of van der Waals contacts with a set of conserved hydrophobic residues (Figure 2b and Figure 3c). The cofactor is further H-bonded (both directly and through water mediated interactions) with the backbone of the penultimate C-terminal residue (Figure 3c). The C-terminal segment (residues 261–264 in *Ec*TS) closes on the active site after cofactor binding, sealing the cavity. The external portion of the active site is highly variable among bTSs, inducing slightly different orientations of the terminal glutamate moiety of the cofactor, which entails mainly water-mediated interactions in this area. Lys48 in *Ec*TS is the only conserved residue that characterizes this site. Lys48 forms a water-mediated interaction with the cofactor glutamate moiety, which has been proved to be important during the enzyme catalysis [29].

The residues responsible for substrate binding are highly conserved also in the human enzyme (Figure 2b). However, the set of hydrophobic residues composing the cofactor site is altered in hTS by the presence of Asn112 that replaces a tryptophan residue (Trp83 in *Ec*TS) shared by bTSs (Figure 2b and Figure 3d). The presence of unshared residues in the active site of bacterial and human enzymes is of key importance for the development of selective bTS inhibitors.

### 2.2. Latest Updates on the TS Catalytic Mechanism

**Scheme 1:**
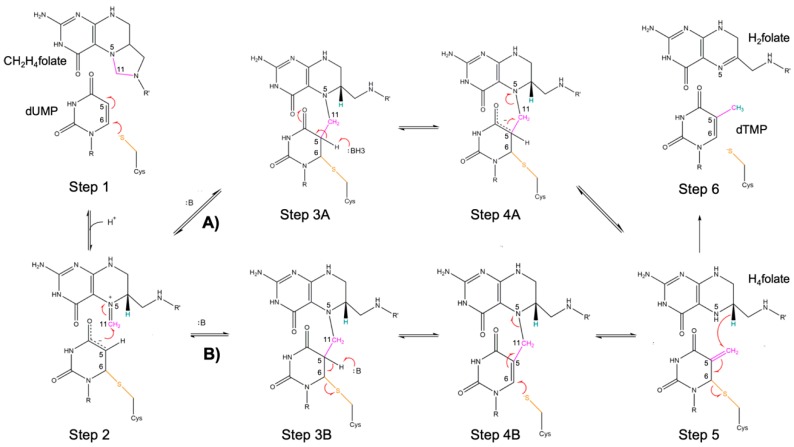
Proposed mechanism for the TS catalyzed reaction. The traditional (**A**) and the recently revised (**B**) paths for the central part of the reaction are displayed. R = 2′-deoxyribose-5′-monophosphate; R’ = *p*-benzoyl-amino-l-glutamic acid. Adapted with permission from Kholodar, SA & Kohen A. Noncovalent Intermediate of Thymidylate Synthase: Fact or Fiction? *J. Am. Chem. Soc.* **2016**, *138*, 8056–8059 [3]. Copyright 2016 American Chemical Society.

TS catalyzes the reductive methylation of dUMP to dTMP, using C_2_H_4_folate as co-substrate (Scheme 1) [1,30]. The catalytic cysteine (Cys146 in *Ec*TS; Figure 2b) is fundamental to activate the substrate and to attract the methylene moiety according to an established mechanism (Steps 1 and 2, Scheme 1) [3,31]. The thiol of the catalytic cysteine attacks the uracil C6 of dUMP leading to the formation of a covalent adduct (Step 1) [3,31]. The uracil C5 is thus activated to attack the imine carbon C11 of the cofactor (resulting from the aperture of the 5-membered ring of C_2_H_4_folate) forming the ternary adduct (Step 2). The mechanism of methyl and hydride transfer from the cofactor has been recently revised on the basis of new computational and experimental evidences [3,32,33,34,35,36]. According to the traditional mechanism (Path A), the transfer of the proton from the uracil C5 generates a covalent enolate intermediate (Step 3A). This stage is followed by the release of H_4_folate (through Hoffman elimination, Step 4A). On the other hand, in the new mechanism (path B) the proton abstraction leads to the cleavage of the covalent bond anchoring the uracil C6 to the cysteine thiol (Step 3B). Subsequently, the intermediate undergoes 1–3 SN_2_ reaction, leading to the regeneration of the covalent bond to the catalytic cysteine (Step 4B). The two mechanisms converge in the formation of the exocyclic methylene intermediate, that undergoes a concerted hydride transfer and cleavage of the C-S bond (1–3 SN_2_ reaction, Step 5), preceding the formation of the products (Step 6). 

### 2.3. Asymmetry and “Half-Site Reactivity” in bTSs, a Still Open Debate

**Figure 4:**
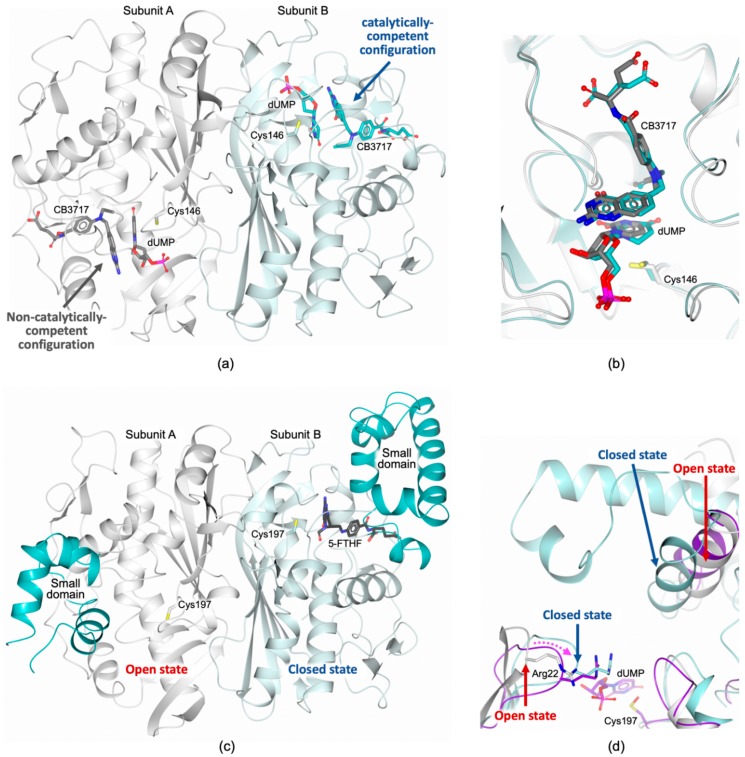
(**a**) Cartoon representation of *Ec*TS I264Am homodimer (Subunits A and B are colored white and light turquoise, respectively; dUMP and CB3717 are shown in sticks, color coded according to the subunit; PDB id 6CDZ [19]). In the active site of Subunit B, the substrate and the cofactor-like inhibitor CB3717 are correctly aligned to mimic a catalytically-competent configuration. On the contrary, in the partner protomer the two molecules assume a non-catalytically-competent configuration. The induced asymmetry, generated in the *Ec*TS dimer, supports the half-site reactivity of the enzyme. (**b**) Active site view of the superimposition between the catalytically-competent configuration and the non-catalytically-competent configuration of *Ec*TS I264Am homodimer. (**c**) Cartoon representation of the *Ef*TS homodimer (Subunits A and B are colored white and light turquoise, respectively, the SD is colored turquoise in both subunits; PDB id 3UWL [16]). The *Ef*TS homodimer is highly asymmetric. Subunit B is in the closed conformation, having the catalytic cavity fully formed, whereas the partner protomer is in the open form, showing a widely unstructured active site. The cofactor analogue 5-formyl-tetrahydrofolate, 5-FTHF (in sticks, black carbons), is entrapped in the active site of Subunit B (**d**) Active site view of the superimposition between the open (white cartoon and carbons) and the closed (light turquoise cartoon and carbons) states of *Ef*TS (PDB id 3UWL [16]) and the *Ef*TS–dUMP complex (purple cartoon and carbons; Cys197 is modified as S-oxycysteine; PDB id 6QYA [41]). dUMP binding in open-state subunits induces the closure of the substrate site (indicated by a dashed pink arrow), whereas cofactor binding is required to fully structure the catalytic cavity. The movement of loop 18–26, determining the closure of the substrate site, is evidenced by the shift of Arg22 (in sticks).

Several multi-subunit enzymes show the phenomenon of “half-site reactivity”. Strictly speaking, half-site reactivity occurs when the reaction with a substrate shows a stoichiometry equal to one-half the number of identical subunits in the multimer. Half-site reactivity represents a form of negative cooperativity between the protomers, in which binding of the substrates in one catalytic site prevents catalysis in the partner subunit. Among bTSs, *Ec*TS is the most widely studied, whereas very little is known about TSs from other pathogenic bacteria. The presence of half-site reactivity in *Ec*TS, was suggested by kinetic studies, differential scanning and isothermal titration calorimetry and fluorescence quenching experiments [29,30,37,38]. Nonetheless, recent studies on *Ec*TS have provided evidence of minimal or absent negative cooperativity in substrate and cofactor binding to both catalytic sites [18,39]. Isothermal titration calorimetry (ITC) experiments have shown that the binding of substrate and cofactor occurs in both enzyme subunits with essentially no cooperativity (only a minor effect is observed in a temperature-dependent manner) [18]. On the other hand, NMR studies have evidenced a tiny, but detectable, extent of negative cooperativity for substrate and cofactor binding in the two *Ec*TS protomers [18,39]. On the basis of these investigations, it has been proposed that *Ec*TS is characterized by a “silent” or “isoergonic” allostery towards dUMP and cofactor binding (meaning that the two binding events have similar affinities but differ in ΔH, ΔS, and/or ΔC_p_) [18,39]. NMR studies have also highlighted a significant contribution of the TS dimer interface in inter-subunit communication [39,40]. Meaningful chemical shift perturbations are observed across the dimer upon the population of both dUMP sites, whereas only local changes are reported when dUMP binds to only one site. The widespread perturbations induced by the second dUMP binding event indicates inter-subunit communication between the two substrate sites (and the two catalytic cavities). These perturbations involve the interface area covering the segment 146–153, Ala132, and residues 198–199 [40]. Therefore, any cooperativity is likely due to long-range, cross-interface effects, occurring upon binding of dUMP in the second site once the first is already populated [39,40]. Very recently, new evidence has been reported on *Ec*TS, upon investigating the *C*-terminal deletion variant I264Am (lacking the *C*-terminal Ile264) [19]. In TS enzymes, the *C*-terminal segment (residues 261–264 in *Ec*TS) rearranges, following the formation of the ternary adduct with substrate and cofactor, closing the cavity and contributing to stabilize the active site during catalysis. The structure of *Ec*TS I264Am shows dimer asymmetry due to different configurations of the catalytic cavities both populated by dUMP and the cofactor analogue inhibitor CB3717 (PDB id 6CDZ [19]; Figure 4a). Substrate and inhibitor are correctly aligned to mimic a catalytically-competent configuration only in the active site of one protomer (Figure 4a,b). On the other hand, the two compounds result slightly displaced in the partner subunit adopting a non-catalytically-competent configuration (Figure 4a,b). This observation supports half-site reactivity for *Ec*TS (Figure 4a). In the structure of *Ec*TS I264Am, changes are observed in the interface area, suggesting that they mediate the inter-subunit communication and regulate half-site reactivity. The most evident change concerns Phe149, facing itself on the partner subunit, for which increased sidechain flexibility is observed in *Ec*TS I264Am asymmetric dimers [19]. All together these evidences suggest that in *Ec*TS both active sites can be concomitantly populated by substrate and cofactor, but the extent of negative cooperativity at the basis of the half-site reactivity is still controversial [18,19,39,40]. However, all studies agree on the prominent role played by the dimer interface on inter-subunit communication. NMR and X-ray crystallographic studies converge on the involvement of the interface segment extending from the catalytic Cys146 to Tyr153 in inter-subunit communication [19,40]. The existence of analogous mechanisms in TSs of other pathogenic bacteria is still unknown.

In bTSs, the SD is highly variable in extension and structure, indeed *Ef*TS and *Sa*TS are characterized by a more extended SD, including an additional segment of 50 amino acids (Figure 2b). This can induce significant differences in asymmetric ligand binding and half-site reactivity among bTSs. The structural characterization of *Ef*TS has highlighted huge differences between the enzyme protomers, resulting in highly asymmetric enzyme homodimers (PDB id 3UWL [16]; Figure 4c). One subunit adopts the so-called closed conformation, in which the catalytic cavity is fully formed and the SD is structured, whereas the partner protomer is in the open form, showing a widely unstructured active site and SD (Figure 4c) [16]. Despite the structure of *Ef*TS suggests that it could be a half-site reactive enzyme, its cooperativity profile has still to be fully elucidated. Recent structural investigations have shown that substrate binding in the open-subunit of *Ef*TS induces the closure of the dUMP site, whereas the rest of the catalytic cavity keeps the open conformation (PDB id 6QYA; Figure 4d) [41]. Therefore, it has been proposed that the open/closed transition occurs in *Ef*TS as biphasic process in which dUMP binding triggers the closure of the substrate site whereas cofactor binding is required to fully structure the catalytic cavity (Figure 4d). In the *Ef*TS-dUMP complex asymmetric substrate binding is consistently observed. On the other hand, the structural characterization of *Sa*TS in complex with dUMP (*Sa*TS; PDB id 4DQ1, unpublished research) shows symmetric ligand binding, having both active sites populated by the substrate. Despite *Ef*TS and *Sa*TS have SDs of similar extension, they display different behaviors, suggesting that it is not possible to delineate common mechanisms for bTSs. Therefore, the recent evidences on *Ec*TS half-site reactivity and inter-subunit communication cannot be directly extended to all bacterial enzymes. The issues of asymmetric ligand binding and half-site reactivity in bTSs of other pathogenic bacteria remain largely unknown and are worthy of careful investigation.

## 3. Flavin-Dependent Thymidylate Synthases (FDTSs)

### 3.1. Structural Insights into FDTSs from Human Pathogens

**Figure 5:**
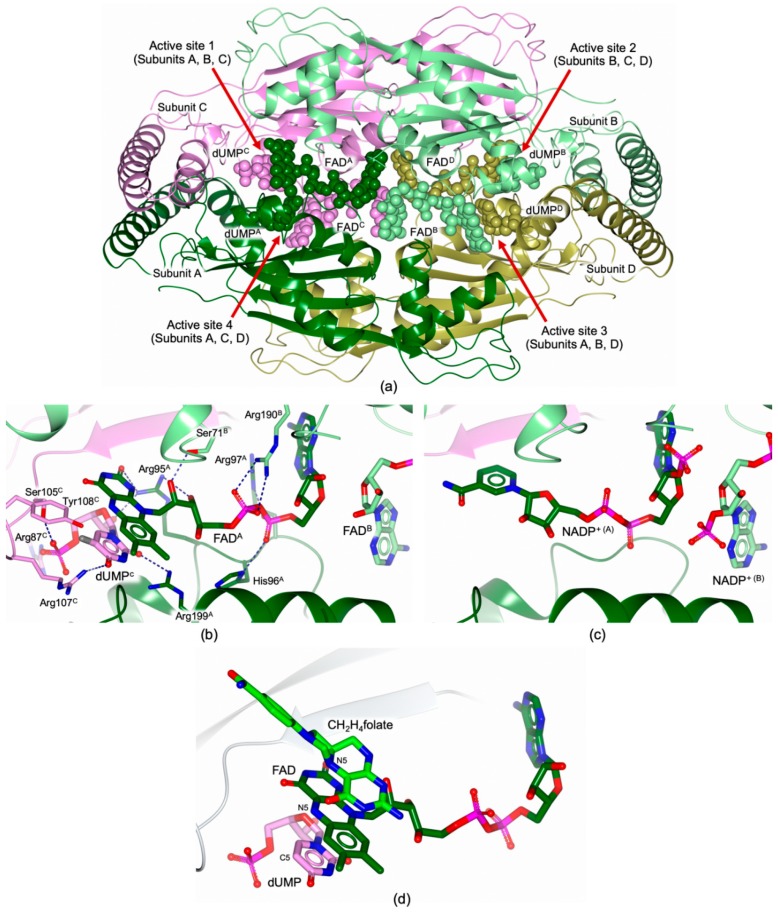
(**a**) Cartoon representation of the FDTS homotetramer (*Mtb*FDTS; Subunit A is colored dark green, B light green, C pink, and D gold; PDB id 3GWC [44]). Subunits contributing to each active site (numbered from 1 to 4) are illustrated in the figure. The Active site 1, composed of residues belonging to Subunits A, B and C, is used for structural description. FAD and the substrate analogue FdUMP are represented in spheres (of arbitrary radius, carbon atoms are color-coded according to the parent subunit). (**b**) H-bonds (blue dashed lines) of FAD and FdUMP (in sticks, carbon atoms are color-coded according to the parent subunit) with residues shared between *Mtb*FDTS and *Hp*FDTS (the *Mtb*FDTS sequence numbering is reported in the picture; PDB id 3GWC [44]). The Active site 1, composed of residues belonging to Subunits A, B and C, is used for structural description in Panels b and c. (**c**) NADP^+^ (in sticks, carbon atoms are color-coded according to the parent subunit) binding mode in the complex with *Mtb*FDTS (PDB id 2GQ2 [45]). (**d**) Active site view of the quaternary complex *Tm*FDTS (white cartoon)–FAD (in sticks, dark green carbons)–dUMP (in sticks, pink carbons)–CH_2_H_4_folate (in sticks, green carbons) (PDB id 4GT9 [46]). The isoalloxazine moiety of FAD is sandwiched between the dUMP uracil and the pteridine of CH_2_H_4_folate. The pteridine N5 of CH_2_H_4_folate is perfectly aligned with both the flavin N5 of FAD and the uracil C5 of dUMP, configurating the active site to allow the FAD-mediated methyl transfer from CH_2_H_4_folate to dUMP (according to the revised reaction mechanisms described in Section 3.2). To facilitate the comparison, Panels b, c, and d have been generated using the same orientation of the FDTS active site.

The structural characterization of FDTSs from human pathogenic bacteria is limited to the enzymes from *Mycobacterium tuberculosis* (*Mtb*FDTS; PDB id 2AF6 [42]) and *Helicobacter pylori* (*Hp*FDTS; PDB id 3N3Y [43]). FDTS are homotetrameric proteins, with four identical subunits forming extensive interactions (Figure 5a). Each subunit shows a complex fold, characterized by a central α/β domain flanked by two helical domains, known as top and bottom domains. The central domain consists of a four-stranded antiparallel β-sheet, flanked on one side by three α-helices. The four active sites are located at the interface between three neighboring monomers, having residues from all three subunits that contributes to create the catalytic cavity (Figure 5a; subunits contributing to each active site are illustrated in the figure; Active site 1, composed of residues belonging to Subunits A, B and C, is used for the structural description) [42,43,44]. FAD acquisition by FDTS was reported to occur inside cells, during the expression of the protein. Therefore, the purified samples already included the cofactor in its oxidized form as indicated by the peculiar yellow colorization of the protein solutions [42,43]. Within each subunit, FAD adopts an extended conformation, covering a total surface area of ~490 Å^2^ [42,43]. The AMP adenine moiety of FAD is buried inside a deep pocket, whereas the AMP ribose faces the ribose of the FAD bound to a neighboring subunit (ribose of FAD^A^ and FAD^B^, belonging to Subunits A and B, respectively, in Figure 5b; FAD^A^ is considered for the present description). The phosphate group of AMP and the ribityl and phosphate moieties of flavinmononucleotide (FMN), belonging to FAD^A^, form H-bonds with the residues of the highly conserved FDTS motif RHR of Subunit A (residues 95^A^–97^A^ in *Mtb*FDTS, Figure 5b; corresponding to 74^A^–76^A^ in *Hp*FDTS). These moieties are also H-bonded with two further conserved residues belonging to Subunit B (Ser71^B^ and Arg190^B^ in *Mtb*FDTS, Figure 5b; corresponding to Ser50^B^ and Arg165^B^ in *Hp*FDTS). Inside the active site, the FAD^A^ isoalloxazine ring is H-bonded with the first arginine of the conserved RHR motif (Arg95^A^ in *Mtb*FDTS, Figure 5b; corresponding to Arg74^A^ in *Hp*FDTS) and with the backbone of a residue belonging to the C Subunit (Gln103^C^ in *Mtb*FDTS and Val82^C^ in *Hp*FDTS; interaction not shown in Figure 5b). The flavin ring of FAD^A^ is stacked with the pyrimidine of dUMP^C^. The complex of *Mtb*FDTS with the substrate analogue FdUMP (PDB id 3GWC [44]) is shown in Figure 5b. FdUMP retains the same binding mode of dUMP in the *Hp*FDTS-dUMP complex (PDB id 3N3Y [43]). Notably, the dUMP^C^ C5 is aligned to the flavin N5 of FAD^A^, separated by a short interatomic distance (<3.5 Å). The alignment of these two atoms is fundamental during the FDTS catalyzed reaction. According to the revised reaction mechanism (detailed in Section 3.2), the methyl is transferred from CH_2_H_4_folate to the FAD flavin N5, that subsequently donates it to the dUMP C5 allowing the formation of the product dTMP. In the catalytic cavity, the dUMP substrate (dUMP^C^) is further stabilized by the interactions with a set of conserved residues (Arg95^A^ and Arg199^A^, Arg87^C^, Ser105^C^ and Arg107^C^ in *Mtb*FDTS, Figure 5b; corresponding to Arg74^A^, Arg174^A^, Arg66^C^, Ser84^C^ and Arg86^C^ in *Hp*FDTS). The dUMP^C^ uracil C5 and the FAD^A^ flavin N5 are shielded from the solvent by a shared tyrosine (Tyr108^C^ and Tyr87^C^ in *Mtb*FDTS and *Hp*FDTS, respectively), whose phenyl moiety forms a “lid” over the reactive positions involved in the methyl transfer (Figure 5b).

The structure of *Mtb*FDTS was reported also in complex with the second cofactor NADP^+^ that occupies the same site of FAD and adopts an analogous binding mode (PDB id 2GQ2 [45]; Figure 5c). The nicotinamide of NADP^+^ is accommodated inside the catalytic cavity in which it replaces the isoalloxazine system of FAD (Figure 5c) [45]. It is worth noting that attempting to obtain the quaternary complex *Mtb*FDTS–FAD–BrdUMP–NADP^+^ by co-crystallization of the enzyme with both cofactors and the substrate analogue BrdUMP, provided the structure of the binary complex *Mtb*FDTS–NADP^+^ [45]. The mechanistic explanation of this behavior is not obvious, since it has been postulated that the regeneration of the reduced flavin during the FDTS catalyzed reaction is mediated by NADPH. Therefore, they should both bind to the enzyme during this stage. The structural characterization of FDTS in complex with both FAD and NADPH can provide key information on the mechanism of reduced flavin regeneration which has still to be fully elucidated.

Structural information concerning the CH_2_H_4_folate binding mode in *Mtb*FDTS and *Hp*FDTS is not available. However, the FDTS-CH_2_H_4_folate complex has been determined for *Thermotoga maritima* FDTS (*Tm*FDTS) [46]. The structure of the quaternary complex *Tm*FDTS–FAD–dUMP–CH_2_H_4_folate (PDB id 4GT9 [46]) shows the isoalloxazine moiety of FAD sandwiched between the dUMP uracil and the pteridine of CH_2_H_4_folate (Figure 5d). In the structure, the pteridine N5 of CH_2_H_4_folate is perfectly aligned with both the flavin N5 of FAD and the uracil C5 of dUMP. This configuration of the active site allows the FAD-mediated methyl transfer from CH_2_H_4_folate to dUMP in accord with the revised reaction mechanism described in Section 3.2.

### 3.2. FDTS Reaction Mechanism

**Scheme 2:**
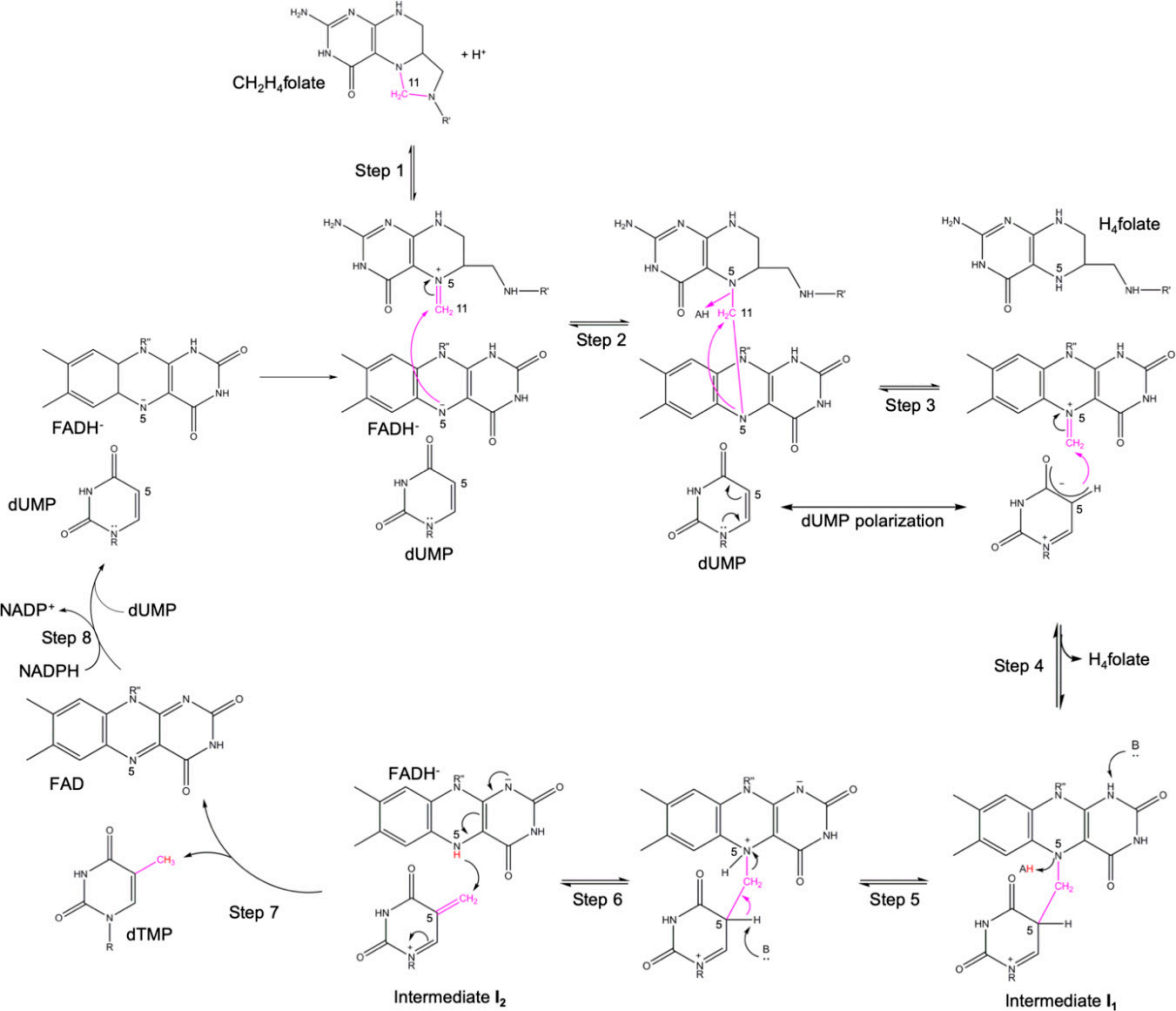
Recently proposed reaction mechanism for FDTSs [4]. R = 2′-deoxyribose-5′-monophosphate; R’ = *p*-benzoyl-amino-l-glutamic acid; R” = adenosine-5′-pyrophosphate-ribityl. Adapted from Reference 4 [4].

The combined results of mass spectrometry, NMR and X-ray crystallographic studies on FDTSs have led Kohen and coworkers to formulate a new reaction mechanism for this class of enzymes, reported in Scheme 2 [4]. The proposed multistep process for FDTSs starts with the protonation of C_2_H_4_folate (Step 1), leading to the formation of a Schiff base, that is activated to transfer the methyl moiety to the flavin N5 of the reduced cofactor FADH (Step 2). In the resulting covalent adduct, the protonation of the folate N5 is mediated by an unknown species (Step 3). Once the methyl moiety is transferred on the FAD cofactor, a new Schiff base is formed. The Schiff base can react with the polarized dUMP (Step 4) to form a covalent intermediate (Intermediate I_1_) in which the methyl group bridges the FADH N5 and the dUMP C5. The generation of the intermediate I_1_ is accompanied by the release of H_4_folate from its site (Step 4). The intermediate I_1_ undergoes protonation on the flavin N5 by an unknown species (Step 5) which precedes the abstraction of the proton from the dUMP C5, still by an uncharacterized actor (Step 6). The deprotonation of the C5 induces the transfer of the methylene group on dUMP (Step 6), leading to the formation of the Intermediate I_2_. I_2_ undergoes a redox reaction in which flavin is oxidized at the expenses of the nucleotide that is reduced to dTMP (Step 7) and subsequently released from its site. The reduced flavin is regenerated by the oxidation of NADPH to NADP^+^ in a second redox reaction (Step 8).

## 4. Inhibitors of TSs and FDTSs from Human Pathogenic Bacteria

### 4.1. Inhibitors of Bacterial TS from Human Pathogens

**Figure 6:**
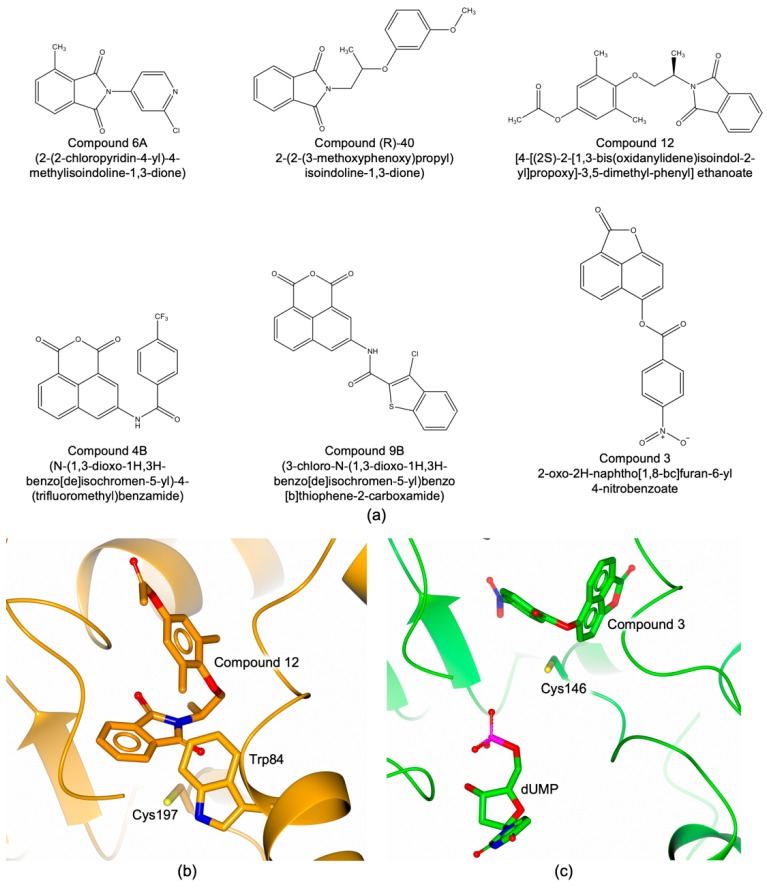
(**a**) Chemical structure of bTS inhibitors. (**b**) Binding mode of Compound **12** (in sticks, orange carbons) within the active site of *Ef*TS (orange cartoon and carbons) (PDB id 4O7U [50]). (**c**) Binding mode of Compound **3** (in sticks, green carbons) within the active site of *Ec*TS (green cartoon and carbons; dUMP in sticks) (PDB id 4LRR [52]).

The main difficulty to develop bTS inhibitors is related to selectivity. The active sites of human and bacterial TSs are highly conserved, the main difference being one unshared residue in the cofactor binding site. Furthermore, variable conformational changes have been characterized in bTSs that are different from those observed in the human enzyme. bTSs shift between open and closed conformations involving structural rearrangements that are peculiar of each bacterial enzyme [16,19,39]. However, hTS switches between the active and inactive conformations, by shifting the catalytic cysteine from the catalytic cavity to the dimer interface [47,48]. In the last decade, different molecular classes have been investigated to selectively target TSs of human pathogenic bacteria. *In silico* studies combined with structural investigations led to the identification of some phtalimide derivatives as selective bTS inhibitors [49,50]. Compounds **6A** and **(*R*)-40** (Figure 6a) resulted effective towards *Ef*TS (K_i_ of 7.0 μM and 13.0 μM, respectively), without affecting the human enzyme [49,50]. The structure of *Ef*TS in complex with a representative phtalimide derivative showed that Compound **12** (Figure 6a, PDB id 4O7U [50]) populates the cofactor site regardless the binding of the substrate (Figure 6b). This configuration is unusual in TS, since cofactor-like inhibitors normally populate their site in presence of dUMP (ternary complexes). The six-membered aromatic ring of phtalimide forms a close van der Waals contact with the *Ef*TS Trp84 (Figure 6b). This residue, shared among bTSs, is replaced by an asparagine (Asn112) in human TS (Figure 3d), providing basis to explain the selectivity profile of these molecules.

Phenolnaphthalein derivatives were also proposed as selective bTS inhibitors [51]. Compounds **4B** and **9B** (Figure 6a) showed relevant selectivity, being active towards *Ec*TS (K_i_ of 6.4 μM and 6.5 μM, respectively) and completely inactive against hTS [51]. Attempting to further improve this class of compounds through the generation of naphthofuranon derivatives resulted in an almost complete loss of selectivity, being also active towards hTS [52]. The structure of *Ec*TS obtained in complex with a representative member of this series, revealed that Compound **3** (Figure 6a, PDB id 4LRR) binds inside the catalytic cavity inducing the rotation of dUMP outside the substrate binding pocket (Figure 6c) [52].

*In vitro* analysis on pyrimidine-5-carbonitrile derivatives [53] and on the ruthenium-based complex [(C_6_H_6_)RuL(*N*,*N*)Cl] [54] reported antimicrobial activity on *S. aureus* and other human pathogenic bacteria. *In silico* studies have identified them as potential *Sa*TS inhibitors [53,54]; however no experimental evidences have proven their activity towards *Sa*TS.

**Figure 7:**
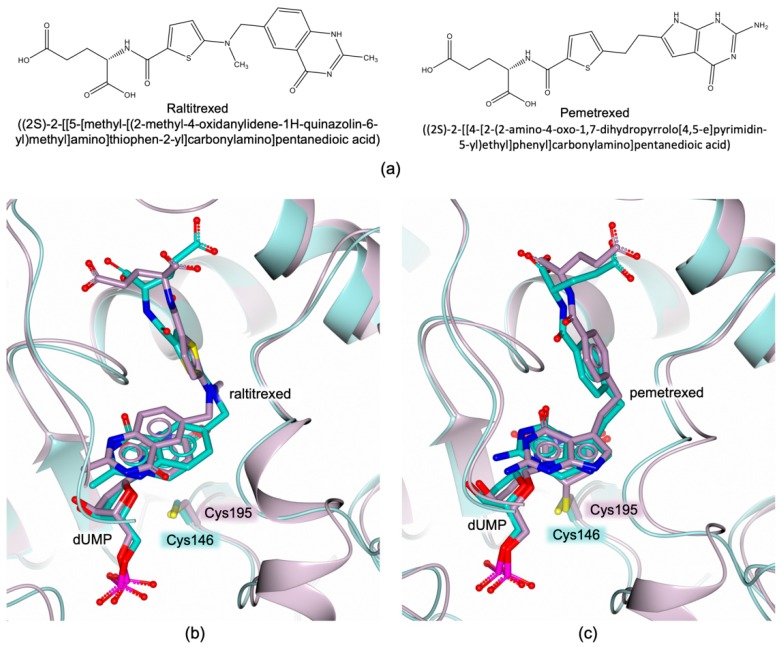
(**a**) Chemical structures of raltitrexed and pemetrexed. (**b**) Active site view of the superimposition of the complexes *Mtb*TS–dUMP–raltitrexed (pale turquoise cartoon and carbons; PDB id 4FOX; unpublished research) and hTS–dUMP–raltitrexed (lilac cartoon and carbons; PDB id 5X5Q [48]). (**c**) Active site view of the superimposition of the complexes *Mtb*TS–dUMP–pemetrexed (pale turquoise cartoon and carbons; PDB id 4FQS; unpublished research) and hTS–dUMP–pemetrexed (lilac cartoon and carbons; PDB id 1JUJ [55]).

Recently, the structures of *Mtb*TS in complex with raltitrexed and pemetrexed (Figure 7a), two known hTS inhibitors, have been reported in the PDB (PDB ids 4FOX and 4FQS, respectively; unpublished research). The comparison between the structures *Mtb*TS–dUMP–raltitrexed and hTS–dUMP–raltitrexed (PDB id 5X5Q [48]) shows the inhibitor bicyclic system in two different orientations inside the catalytic cavities of the two enzymes (Figure 7b). However, the binding mode of pemetrexed is almost conserved in the active sites of *Mtb*TS and hTS (Figure 7c). Differences in the ligand binding mode between *Mtb*TS and hTS are exploitable for the development of selective bTS inhibitors.

### 4.2. FDTS Inhibitors towards Human Pathogenic Bacteria

Microbial FDTSs have no structural homology with hTS, being highly divergent in the configurations of the active sites and in their catalytic mechanisms. Therefore, FDTSs represent promising targets for the development of new antimicrobial drugs.

**Figure 8:**
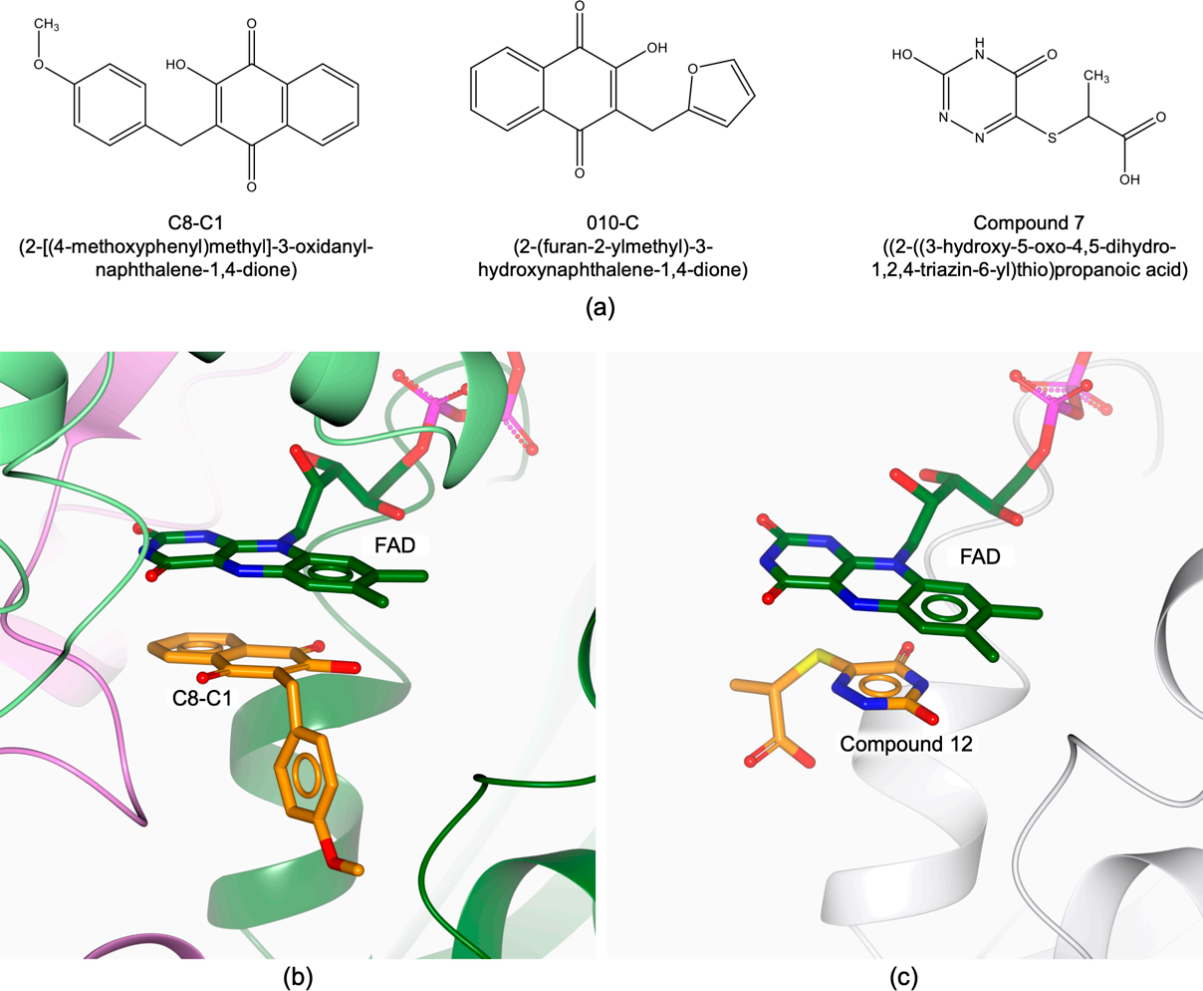
(**a**) Chemical structures of FDTS inhibitors. (**b**) Binding mode of the NQ derivative **C8**-**C1** (in sticks, orange carbons) in the active site of *PBC*V1FDTS (in cartoon, Subunit A is colored dark green, B light green, and C pink; FAD in sticks, dark green carbons; PDB id 4FZB [56]). (**c**) Binding mode of Compound **12** (in sticks, orange carbons) in the active site of *Tm*FDTS *(*white cartoon; FAD in sticks, dark green carbons; PDB id 5CHP [58]).

Natural compounds are an important source to identify new chemical scaffolds. A library of more than 2300 natural compounds was screened towards FDTSs from various pathogenic bacteria (including *Hp*FDTS, *Mtb*FDTS, and *Chlamydia trachomatis* FDTS, *Ct*FDTS) [56]. In this study, 1,4-naphthoquinone (NQ) derivatives were identified as FDTS inhibitors. Subsequent studies on the NQ derivative **C8**–**C1** (Figure 8a) showed that it is a potent inhibitor of *Hp*FDTS (estimated K_i_ of 367 nM), displaying meaningful antimicrobial activity on *H. pylori* (MIC 10 μg mL^−1^) [57]. The structure of **C8**-**C1** in complex with the FDTS from *Paramecium bursaria chlorella* virus (*PBC*V1 FDTS; having an almost conserved active site with respect to bacterial FDTSs) showed that the compound occupies the catalytic cavity, replacing the dUMP uracil (Figure 8b; PDB id 4FZB [56]).

NQ derivatives were further developed, resulting in two compounds with improved potency towards *Hp*FDTS [57]. Compound **010**-**C** (Figure 8a) was the most potent *Hp*FDTS inhibitor, having a K_i_ in the low nano-molar range (K_i_ of 28 nM) [57]. These improved NQ derivatives resulted also effective towards *H. pylori* (MIC ranging from 0.625 to 10 μg mL^−1^). The three most potent compounds of this series were also investigated *in vivo* using a mouse model for *H. pylori* infection. The compounds were tolerated in mice, but displayed a modest antibacterial effect [57].

In 2016, *in silico* studies on FDTS were combined with structural investigations leading to the identification of a new molecular scaffold exploitable for the development of novel FDTS inhibitors [58]. The binding mode of Compound **7** (Figure 8a, 6% inhibition at 100 μM towards *Mtb*FDTS) was clarified in complex with the FDTS from the non-pathogenic *Thermotoga maritima (Tm*FDTS, PDB id 5CHP [58]). The structure shows that Compound **7** occupies the dUMP site within the catalytic cavity. Biochemical assays performed on hTS, evidenced the ineffectiveness of the compound on this target, indicating this scaffold useful for the development of novel FDTS inhibitors.

Recently, an high-throughput screening has been performed on *Mtb*FDTS, using a library of 40,000 compounds [59]. *In silico* studies have led to the identification of 1,4-benzoxazine derivatives as FDTS inhibitors. Further investigations have proven that Compound **B1**-**PP146** (unreported chemical structure) was the most potent 1,4-benzoxazine derivative of this series. This compound is effective on *Mtb*FDTS (IC_50_ of 0.71 μM), displaying competitive inhibition against CH_2_H_4_folate [59].

### 4.3. dUMP-Like Inhibitors towards Human Pathogenic Bacteria Expressing Both TS and FDTS Enzymes

**Figure 9:**
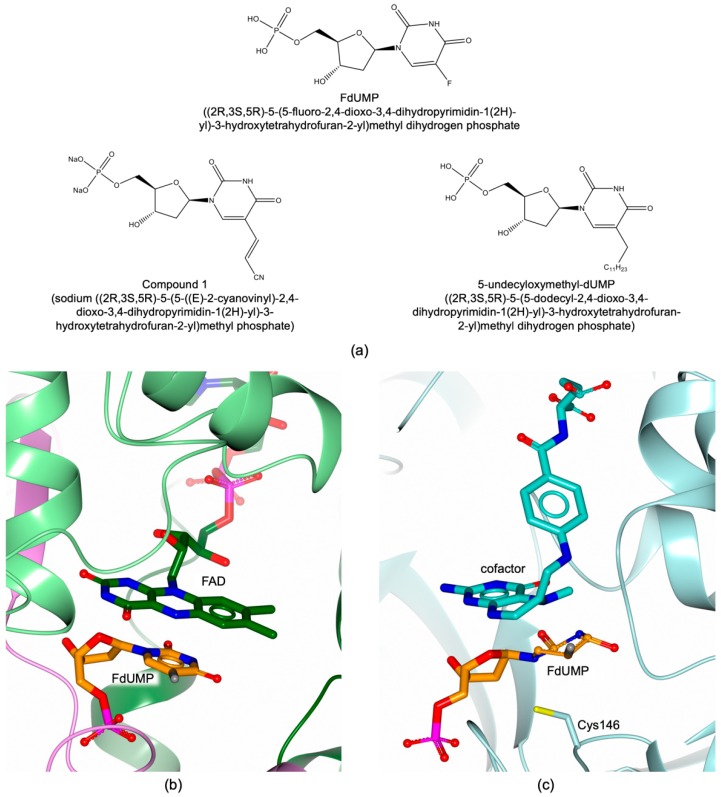
(**a**) Chemical structures of FdUMP and substrate analogue inhibitors. (**b**) Binding mode of FdUMP (in sticks, orange carbons) in the active site of *Mtb*FDTS (in cartoon, Subunit A is colored dark green, B light green, and C pink; FAD in sticks, dark green carbons; PDB id 3GWC [44]). (**c**) Binding mode of FdUMP (in sticks, orange carbons) in the active site of *Mtb*TS (pale turquoise cartoon and carbons; CH_2_H_4_folate in sticks, turquoise carbons; PDB id 4FOG; unpublished research).

*Bacillus anthracis*, *Clostridium botulinum*, and *Mycobacterium* species are examples of human pathogenic bacteria expressing both TS (*thyA* gene) and FDTS (*thyX* gene) enzymes [2]. Studies performed on *Mycobacterium tuberculosis* showed that the *thyX* gene is essential for bacteria survival and its overexpression was observed in multi-drug resistant strains [6,8]. However, the *thyA* gene was reported less essential for *M. tuberculosis* but connected with the resistance to *p*-aminosalicylic acid [6]. The effects of FDTS inhibition on bacteria expressing both methyltransferase enzymes have to be fully elucidated and the potential involvement of TS as metabolic bypass to FDTS inhibition is not excluded. The ability of these enzymes to recognize the same substrate suggested its analogue FdUMP (Figure 9a) as dual TS–FDTS inhibitor [60,61]. Indeed, FdUMP was reported as potent inhibitor of both *Mtb*TS (K_i_ of 2 nM) and *Mtb*FDTS (K_i_ 100 nM) (Figure 9b,c), exhibiting also a remarkable antimycobacterial activity (MIC 3.1 μM) [60,61].

In 2011, a series of 5-substituted-dUMP analogues was probed towards *Mtb*FDTS and *Mtb*TS [62]. Compound **1** (Figure 9a) resulted the most potent *Mtb*FDTS inhibitor (IC_50_ of 0.91 μM), showing no noticeable activity on *Mtb*TS (IC_50_ > 50 μM) [62]. The replacement of dUMP with 6-aza-dUMP in a new series of 5-substituted derivatives induced a drop of their potency towards *Mtb*FDTS (maximal inhibition of 40% at 50 μM), resulting inactive also against *Mtb*TS [63]. Analogously, the replacement of the nucleotide with an acyclic nucleoside phosphonate moiety (ANP derivatives) reduced the activity towards *Mtb*FDTS (maximal inhibition of 43% at 50 μM) [64].

Structure-activity relationship analysis combined with *in silico* studies led to the development of 5-undecyloxymethyl-dUMP (Figure 9a), for which an IC_50_ of 8.32 μM against *Mtb*FDTS was reported [65]. On the other hand, no detectable inhibition was observed on *Mtb*TS (up to concentrations of 100 μM) [65]. This compound, together with other derivatives of this series, displayed a significant antibacterial activity (MIC 10–20 μg mL^−1^) towards two mycobacterial strains, the virulent laboratory H37Rv strain and the multi-drug resistant MS-115 [66].

Very recently, a further series of nucleotide analogues has been investigated [67]. Various 5-modified 6-aza- and 2-thio-6-aza-2′-deoxyuridine derivatives have shown a significant antibacterial activity on important human pathogens, including *Mycobacterium smegmatis*, *Staphylococcus aureus* and *Pseudomonas aeruginosa* [67]. Docking studies have predicted that they target *Mtb*FDTS. Nonetheless, their activity on *Staphylococcus aureus* and *Pseudomonas aeruginosa*, lacking the *thyX* gene, strongly suggests that these compounds inhibit other bacterial enzymes.

## 5. Conclusions

Methyltransferase enzymes of human pathogenic bacteria represent important targets for the development of new antibiotic drugs. The catalytic processes of TS and FDTS have been recently revised, but their mechanisms of action are not yet fully elucidated. For bTSs, half-site reactivity is still an open debate. Recent insights obtained on *Ec*TS are somehow controversial [18,19,39,40], suggesting that deeper investigations are required to unveil the extent of inter-subunit cooperativity in this enzyme. The TS dimer interface area has been shown to play a major contribution in the inter-subunit communication occurring between the two holoenzyme halves during the catalytic process. The TS dimer interface is crucial for the enzyme activity, but its potential as drug-targetable area is yet unexplored for bacterial enzymes.

FDTSs represent a promising target for development of new antibacterial drugs since they have no human counterpart enzymes. On the other hand, in FDTS, the mechanism of the redox reaction responsible for the regeneration of reduced flavin is not fully elucidated. During this process, flavin is reduced at the expenses of NADPH that is oxidized to NADP^+^. Understanding how FAD and NADPH interact with FDTS during this stage of the catalytic reaction would represent a breakthrough to elucidate the mechanism by which the flavin cofactor is regenerated by these enzymes.

The development of FDTS-targeting molecules has led to the identification of potent inhibitors. Nonetheless, the *in vivo* properties of these compounds have to be improved to achieve suitable drug candidates. Targeting this enzyme is crucial not only in human pathogens relying only on FDTS, but also in those expressing both FDTS and TS enzymes. Although blocking the FDTS activity is pivotal, the expression of TS could provide a metabolic bypass to FDTS inhibition, leading to resistance. The importance of developing bTS inhibitors is further evidenced by the existence of important human pathogens relying only on TS. The high conservation of the active site among bacterial and human TSs constitutes an important issue for the design of selective bTS inhibitors. Relevant steps forward have been recently reported opening new perspectives for the development of effective and selective bTS-targeting drugs. The recent structural evidence acquired on bTSs from human pathogenic bacteria has also highlighted prominent differences in the conformational flexibility among human and bacterial enzymes, yet unexplored for the design of selective bTS inhibitors.

These are promising results; however, more efforts are required to obtain drug candidates targeting bTS and FDTS enzymes.

## Abbreviations

The following abbreviations are used in the manuscript:

dTMP	2′-deoxythymidine-5′-monophosphate
dUMP	2′-deoxyuridine-5′-monophosphate
TS	thymidylate synthase
DHFR	dihydrofolate reductase
FDTS	flavin dependent thymidylate synthase
CH_2_H_4_folate	*N*^5^,*N*^10^-methylene-5,6,7,8,-tetrahydrofolate
H_2_folate	dihydrofolate
H_4_folate	tetrahydrofolate
FAD	flavin adenine dinucleotide
NADPH	nicotinamide adenine dinucleotide phosphate
PDB	Protein Data Bank
